# Iron-Based Metal–Organic Frameworks and Their Polymer Composites for Sustainable Delivery of Herbicides

**DOI:** 10.1021/acsomega.4c07972

**Published:** 2025-02-26

**Authors:** Parimal C. Bhomick, Evdokiya H. Ivanovska, Lila A. M. Mahmoud, Huan V. Doan, Lui R. Terry, Matthew A. Addicoat, Jemma L. Rowlandson, Sebastien Rochat, Valeska P. Ting, Sanjit Nayak

**Affiliations:** † Bristol Composites Institute, Queen’s Building, University of Bristol, University Walk, Bristol BS8 1TR, U.K.; ‡ Department of Chemistry, Nagaland University, Lumami Campus, Lumami, Nagaland 798627, India; § School of Electrical, Electronic and Mechanical Engineering, University of Bristol, Queen’s Building, University Walk, Bristol BS8 1TR, U.K.; ∥ Research School of Chemistry, Australian National University, Canberra, Australian Capital Territory 2601, Australia; ⊥ School of Archaeological and Forensic Sciences, University of Bradford, Bradford BD7 1DP, U.K.; # School of Chemistry, University of Bristol, Bristol, BS8 1TS, U.K.; ∇ School of Civil, Aerospace and Design Engineering, University of Bristol, Queen’s Building, University Walk, Bristol BS8 1TR, U.K.; ○ School of Science and Technology, Nottingham Trent University, Clifton Lane, Nottingham NG11 8NS, U.K.; ▲ School of Engineering Mathematics and Technology, University of Bristol, Bristol BS8 1TR, U.K.

## Abstract

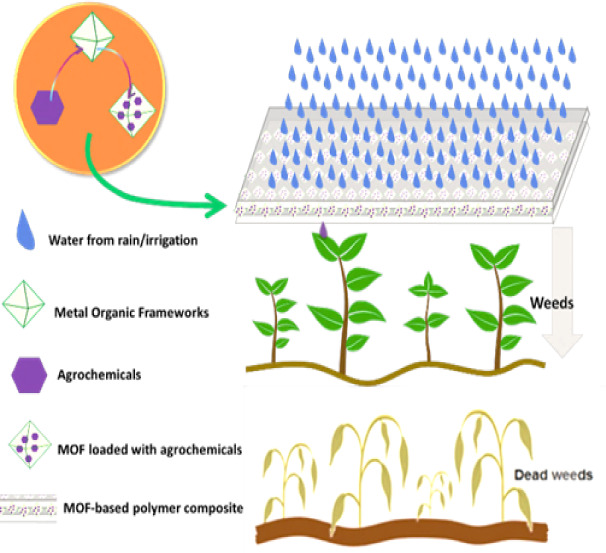

Sustainable agriculture will play a key role in ensuring food security for the rising global population. Controlled and precision delivery of agrochemicals, such as herbicides and pesticides, plays a critical role in sustainable agriculture. Recently, porous metal–organic frameworks (MOFs) have shown promising results for controlled agrochemical delivery. Because of their low toxicity and biocompatibility, iron-based metal–organic frameworks (Fe-MOFs) are highly suitable for applications in agriculture over many other MOFs. In this study, two iron-based MOFs, MIL-101(Fe) and NH_2_-MIL-101(Fe), and their biodegradable polymer composites were studied for controlled herbicide delivery. Two herbicides, 2,4-dichlorophenoxyacetic acid (2,4-D) and 2-methyl-4-chlorophenoxyacetic acid (MCPA), were postsynthetically loaded into these two Fe-MOFs and incorporated into a biodegradable polycaprolactone (PCL) matrix to form composite membranes for ease of handling and delivery. MIL-101(Fe) showed loading capacities of 18.06 and 21.51 wt %, respectively, for 2,4-D and MCPA, while for NH_2_-MIL-101(Fe), the loading capacities for the same herbicides were 26.61 and 23.32 wt %. Despite high loading capacity, both MOFs showed a certain degree of degradation during herbicide loading. The release of 2,4-D and MCPA from MIL-101(Fe) and NH_2_-MIL-101(Fe) and their PCL composites were studied using UV–visible spectroscopy over a nine-day period. NH_2_-MIL-101(Fe) and its PCL composite demonstrated slower and more controlled release profiles of the herbicides compared to MIL-101(Fe) and its composites. The results were also corroborated by computational studies, which showed stronger interactions of the herbicides with NH_2_-MIL-101(Fe).

## 1 Introduction

The use of agrochemicals, such as pesticides and herbicides, is essential in modern agriculture as we race to keep up with global food demand. However, their accumulation in the environment, along with their degradation products, poses a great threat to the environment, human health, and the wider ecosystem. This includes contamination of groundwater and food chains, which can lead to severe health issues such as cancer, neurological disorders, immunological issues, and reproductive defects.^1^ Several pesticides and other agrochemicals have been under scrutiny because of their associated toxicity but remain in use as they are essential for maintaining crop productivity and meeting global food demands.^2−4^ Pesticides are mainly delivered by conventional methods, such as blanket spraying over large vegetation areas. These methods use a very high initial delivery dose (up to 500 L/ha),^5^ and it is estimated that only 0.1% of the applied pesticides reach the intended target. The rest of the dose enters the environment directly through air, water, and soil, directly contributing toward high levels of ecotoxicity.^6−10^ It is therefore essential that a controlled release technology be developed for more targeted and environmentally friendly pesticide delivery. Many approaches, such as chemical degradation, membrane filtration, microbial treatment, and adsorption, have been investigated to address these issues and lessen the environmental impact.^11^ Out of these methods, adsorption has outperformed the others as it offers a simpler and more effective way to extract pesticides.^12−14^ Several elements need to be considered for an effective adsorption process, including the adsorbent’s high porosity, surface area, and interaction with the adsorption sites. In this regard, metal–organic frameworks (MOFs) serve as ideal candidates. Metal–organic frameworks are a class of materials made of metal ions/clusters connected with organic linkers, often forming crystalline 2D or 3D networks presenting permanent porosity. Compared to traditional adsorbents, MOFs offer several advantages, including high surface area, high and tunable porosity, and easy postsynthetic modification for tailored host–guest interactions. Due to their high uptake capacity and selective adsorption, MOFs have been extensively studied for the removal and sensing of agrochemicals.^15^ Moreover, MOFs can serve as delivery platforms for agrochemicals, representing an emerging area of study with growing interest.^11^

Among the plethora of MOFs synthesized to date, iron-based MOFs (Fe-MOFs) stand out as excellent candidates for agricultural applications because of their cost-effectiveness and environmental friendliness, allowing these MOFs to serve dual functions: (a) as hosts for agrochemicals for controlled delivery and (b) as a micronutrient source for plant growth.^11,16^ MIL-101(Fe)^17,18^ is a mesoporous Fe-MOF with pore dimensions of 12 Å × 29 Å and 16 Å × 34 Å, composed of nontoxic components, making it a relatively ecofriendly^16^ and biocompatible^19,20^ MOF suitable for agricultural applications.

However, the crystalline and often powdery form of pristine MOFs including MIL-101(Fe) poses a challenge for their handling, transport, and practical applications. To overcome this barrier, crystalline MOFs can be incorporated into polymer composites. Such hybrid MOF/polymer composites can improve handling, transport, and overall versatility, expanding their scope for potential applications.^21,22^ Polycaprolactone (PCL) was selected as the polymer matrix for this study due to its biocompatibility and nontoxicity.^23^ Its superior mechanical properties,^24^ along with a relatively slow hydrolytic degradation rate compared to other biodegradable polymers like polylactic acid, make PCL an ideal candidate for fabricating polymer composites for agrochemical delivery.^25^

**Scheme 1:**
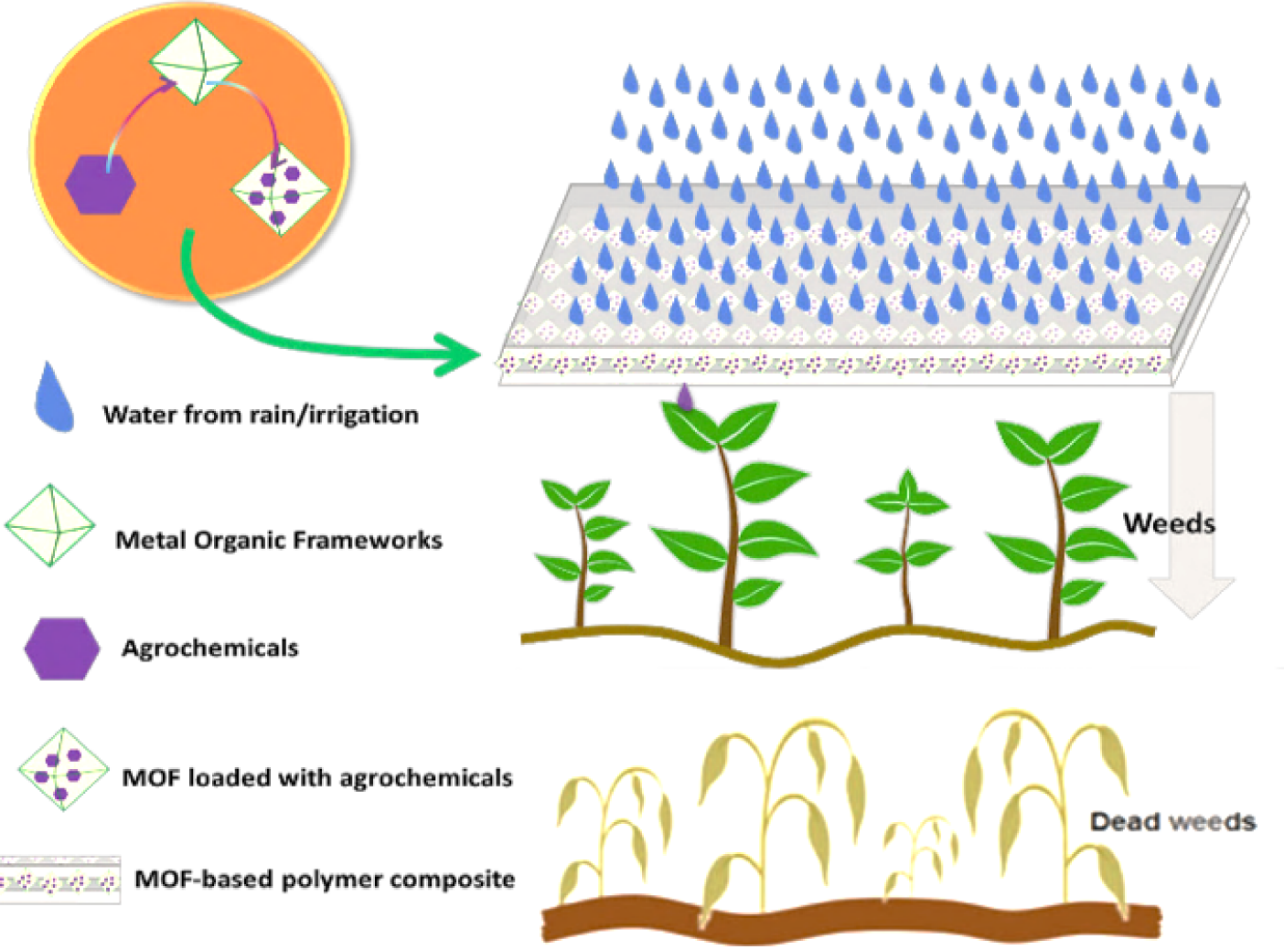
Illustration of MOF-Based Polymer Composite for Delivery of Agrochemicals and Pesticides

In this study, two nontoxic and biocompatible MOFs (MIL-101(Fe) and NH_2_-MIL-101(Fe)) were synthesized and loaded with two herbicides, 2,4-dichlorophenoxyacetic acid (2,4-D) and 2-methyl-4-chlorophenoxyacetic acid (MCPA), as model agrochemicals. These herbicides are widely used for broadleaf weed control in both crop and noncrop lands due to their effective herbicidal activities. Subsequently, these loaded MOFs were integrated into a biodegradable polymer (polycaprolactone) to form MOF-based composite membranes for the controlled release of 2,4-D and MCPA (Scheme 1). As illustrated in Scheme 1, these biodegradable membranes can be activated by irrigation water or rain, releasing agrochemicals at a controlled rate.

This enhances targeted delivery and reduces the overuse of agrochemicals. Due to biocompatibility of the iron-based MOFs and the biodegradability of PCL, the composite would eventually degrade, and the iron from the MOFs can be utilized as a micronutrient replenishment, making the process sustainable.

## 2 Materials and Methods

### 2.1 Material Used

Iron(III) chloride hexahydrate (ACS reagent, 97%), terephthalic acid (98%), 2-amino terephthalic acid (99%), and the herbicides (≥95.0%) were purchased from Sigma-Aldrich, and dimethylformamide and ethanol were purchased from Fisher Chemicals. Polycaprolactone (molecular weight ∼80,000) was purchased from Acros Organics. All chemicals were used as received without any further purification.

### 2.2 Synthesis of MIL-101(Fe)

For the synthesis of MIL-101(Fe), a solvothermal approach was used. 0.412 g (2.48 mmol) of 1,4-benzenedicarboxylic acid and 1.3244 g (4.9 mmol) of FeCl_3_·6H_2_O were dissolved in 30 mL of DMF by sonication for 20 min in a Teflon-lined vial. The mixture was heated to 110 °C at a heating rate of 5 °C/min, maintained at 110 °C for 20 h, and cooled to room temperature at 0.5 °C/min. The product was collected by centrifugation (4500 rpm, 20 min) and washed with DMF and ethanol 3 times to remove unreacted compounds and then dried at 60 °C for 20 h under vacuum. The color of the final product was light orange.

### 2.3 Synthesis of NH_2_-MIL-101-Fe

For the synthesis of NH_2_-MIL-101-Fe, 2-amino terephthalic acid (0.449 g, 2.48 mmol) and FeCl_3_·6H_2_O (1.3244 g, 4.9 mmol) were dissolved in a Teflon-lined vial by sonication at 25 °C for 20 min. The mixture was heated to 110 °C at a heating rate of 5 °C/min, maintained at 110 °C for 20 h, and cooled to room temperature at 0.5 °C/min. The dark brown powder was collected by centrifugation (4500 rpm, 20 min) and washed with DMF and ethanol 3 times to remove unreacted compounds and then dried at 60 °C for 20 h under vacuum.

### 2.4 Preparation of Polycaprolactone-MOF Composites

In a 50 mL round-bottom flask, 200 mg of polycaprolactone polymer (PCL) was allowed to dissolve in 15 mL of chloroform by stirring the mixture for 30 min at 800 rpm. In a 10 mL glass vial, 5 mL of the PCL solution was added to 5 mg of ground MOF powder and stirred for 30 min at room temperature on a magnetic stirrer at 600 rpm. The MOF-PCL solution was cast into a Teflon mold with a circumference of 11.62 cm and was left to dry at room temperature to yield polymer-composite sheets (see Figure S1).

### 2.5 Characterization

Fourier-transformed Infrared (FTIR) spectra were recorded over the range of 600–4000 cm^–1^ using a PerkinElmer Spectrum 100 FTIR spectrometer fitted with a PerkinElmer Universal ATR sampling device. Thermogravimetric analyses (TGA) were carried out using a Q5000IR thermogravimetric analyzer (TA Instruments, USA). Samples (ca. 5 mg) were heated in platinum pans from 30 to 700 °C at 5 °C min^–1^ under a nitrogen purge gas flow of 25 mL min^–1^. SEM images and energy-dispersive X-ray spectroscopy (EDS) data for elemental analysis were collected using an FEI Quanta 400 E-SEM instrument fitted with an Oxford Xplore30 EDS system. The samples were sputter-coated with gold using an Emitech K550 coating system, and the analyses were carried out under vacuum. Information on the specific surface area and internal pore structure was obtained from N_2_ adsorption at 77 K on a Micromeritics 3Flex volumetric gas sorption analyzer. Each material (∼60–80 mg) was degassed prior to the experiment (388 K, ∼8 h, 1 × 10^–6^ mbar). Helium was used for the free-space determination following isothermal data collection. N_2_ and helium were supplied by Air Liquide and of purity 99.999%. Pore volume distribution as a function of pore width was calculated from the N_2_ adsorption data using a density functional theory (DFT) fitting and a cylindrical pore – NLDFT Tarazona Esf = 30 K model. The BET surface area was determined following the procedure outlined in ISO 9277. A Rouquerol correction was applied to the BET fitting to calculate the surface areas. A resultant correlation function of >0.9999 was observed for each material and a positive intercept (Figure S4).

### 2.6 Loading Capacity

Loading capacity was determined using a method reported by dos Reis et al.,^26^ with a slight modification. 5 mg of the loaded MOFs (see Supporting Information for loading of 2,4-D and MCPA into the MOFs) was suspended in 3 mL of distilled water and sonicated for 60 min. After sonication, a 1 mL aliquot of the solution was taken out and diluted with 2 mL of water. The concentration was measured using a UV–vis spectrophotometer at 230 nm for 2,4-D and 227 nm for MCPA. The mass of herbicide released was determined from the volume, and the loading capacity in percentage was calculated using the following equation:
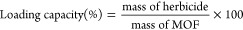


### 2.7 Release Study

For the release study, 5 mg of loaded MOFs and the MOF-PCL composite was submerged into 30 mL of deionized (DI) water at room temperature in a sealed container. 3 mL of the sample was collected from each set at different time intervals; at the same time, the stock was replaced by 3 mL of DI water to maintain the volume at 30 mL. A 1 mL portion of the sample was added to a quartz cuvette with 2 mL of water, and the concentrations were calculated using a UV–vis spectrophotometer at 230 nm for 2,4-D and 227 nm for MCPA.

## 3 Results and Discussion

Two iron-based MOFs, MIL-101(Fe) and NH_2_-MIL-101(Fe), were prepared via a solvothermal method. FeCl_3_ was added to 1,4-benzenedicarboxylic acid and 2-amino-1,4-benzenedicarboxylic acid, respectively, using DMF at 110 °C for 24 h in 30 mL Teflon-lined vials, following a previously reported procedure with a slight modification.^27^ Following solvent exchange and drying, the activated MOFs were loaded with two different herbicides, 2,4-D and MCPA, by stirring the MOFs into the herbicide solution at 600 rpm for 3 days under ambient conditions. These loaded MOFs were subsequently incorporated into biodegradable polycaprolactone membranes via the casting method and were studied for release of the herbicides until 216 h in an aqueous medium. For comparison, MOFs loaded with the herbicides were also investigated in parallel release studies.

### 3.1 Characterization of Fe-MOFs

The MOFs were characterized using PXRD, TGA, FTIR, SEM, and EDS. The PXRD patterns of MIL-101(Fe) and NH_2_-MIL-101(Fe) were compared against the isostructural MIL-101(Cr), calculated from the previously reported single-crystal data.^28,29^ The PXRD pattern of pristine MIL-101(Fe) aligns well with the reported PXRD pattern of MIL-101(Cr). The high-intensity diffraction peaks at 2θ of 9.25, 10.49, 16.63, and 18.08 are also consistent with previously reported results for MIL-101(Fe).^30^ For NH_2_-MIL-101(Fe), two diffraction peaks similar to those of MIL-101(Fe) were observed at 9.36°, 16.86°, and 18.99° in 2θ degrees.^30^ In addition, the sharp peaks indicate good crystallinity of both MIL-101(Fe) and NH_2_-MIL-101(Fe). Similar findings have been reported in the literature, showing the high degree of crystallinity for MIL-101(Fe) and NH_2_-MIL-101(Fe).^27,30−33^ On loading the herbicides, diminished intensity and broadening for the low-angle peaks (such as at 2θ = 11) were observed, which can be due to a change in lattice or degradation of crystallinity as reported in other studies with MIL-101(Fe).^34,35^ The absence of the strong peaks around 2θ = 16 confirms that these peaks are not originating from particulate MCPA or 2,4-D (Figure S2). In the case of the PCL composites, the larger proportion of PCL reflects on the PXRD pattern where the other peaks are masked by the peaks originating from PCL, such as the strong peak at 2θ = 21 (see Figure S2 for PXRD of PCL).

The surface morphology of all the samples was investigated using SEM, corroborating the PXRD results and confirming the presence of the MOF crystalline nature. SEM images in Figure 2 reveal that MIL-101(Fe) crystals have a typical octahedral shape (Figure 2a), whereas the synthesized NH_2_-MIL-101(Fe) crystals have a hexagonal morphology (Figure 2b).^30^ The degradation of crystallinity observed in the powdered XRD was also evident in the morphology of 2,4-D and MCPA-loaded MIL-101(Fe) and NH_2_-MIL-101(Fe) (Figure 2 c–f). This could be caused by dissolution and mechanical stirring during the loading process. The SEM images (Figure 3g–j) and EDS mapping (Figure S3) show a smooth, homogeneous surface of the composites with uniform distribution of the Fe-MOFs.

**Figure 1:**
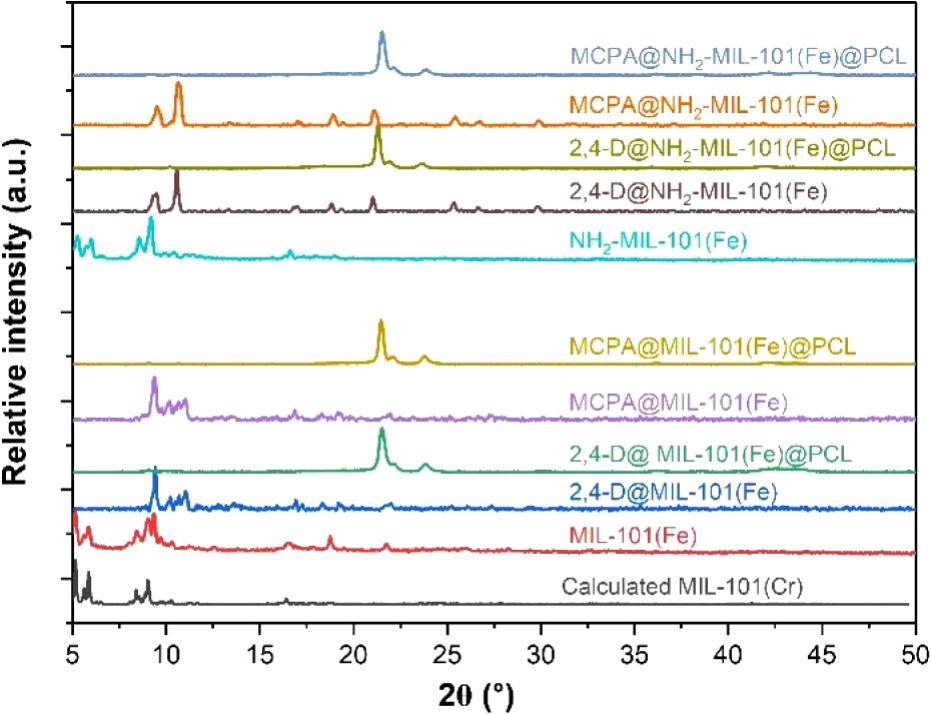
PXRD for calculated MIL-101(Cr) and experimentally measured for pristine and loaded MIL-101(Fe) and NH_2_-MIL-101(Fe) samples and their PCL composites.

**Figure 2:**
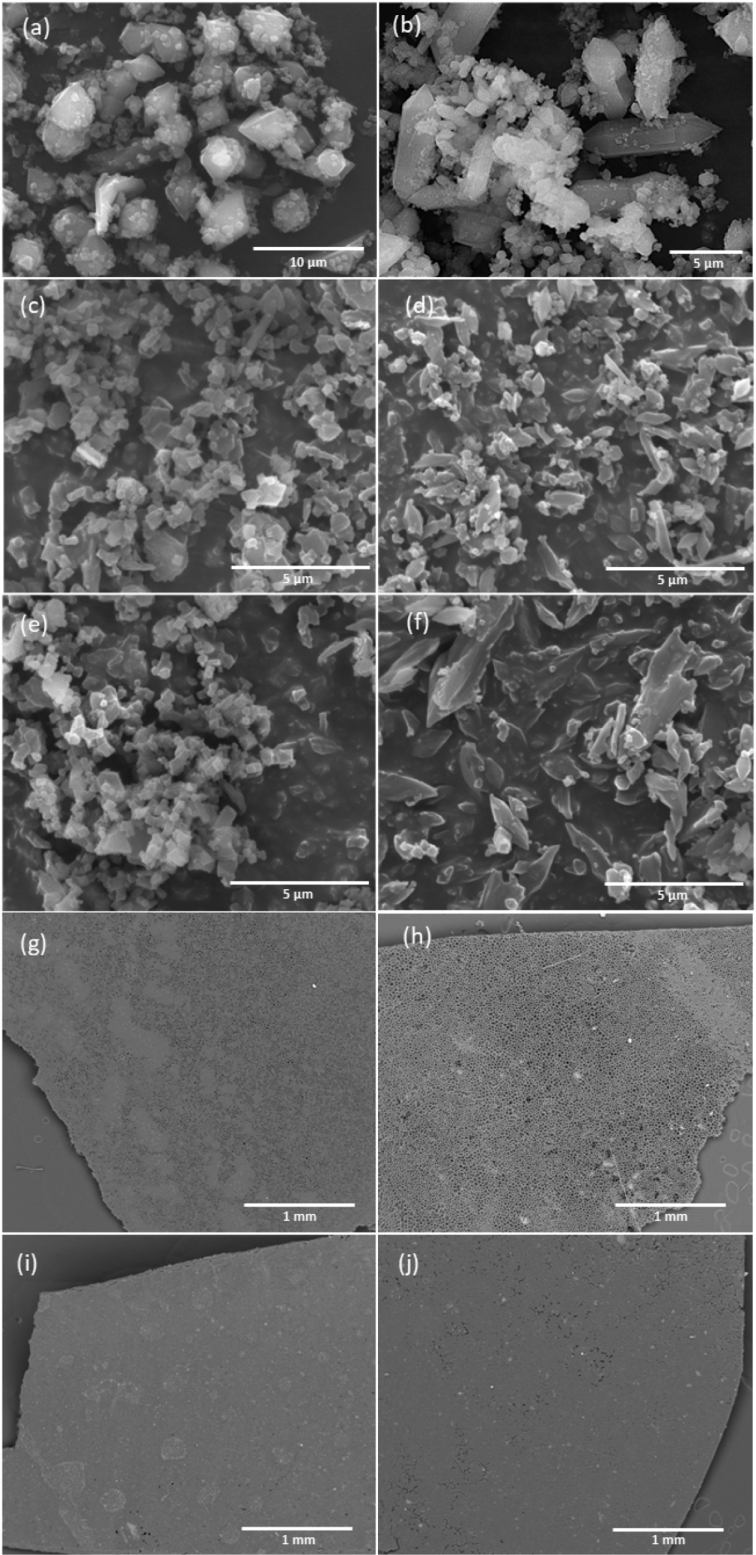
SEM images of (a) MIL-101(Fe), (b) NH_2_-MIL-101(Fe), (c) 2,4-D@MIL-101(Fe), (d) 2,4-D@NH_2_-MIL-101(Fe), (e) MCPA@MIL-101(Fe), (f) MCPA@NH_2_-MIL-101(Fe), (g) 2,4-D@MIL-101(Fe)@PCL, (h) 2,4-D@NH_2_-MIL-101(Fe)@PCL, (i) MCPA@MIL-101(Fe)@PCL, and (j) MCPA@NH_2_-MIL-101(Fe)@PCL [scale bar: (a) 10 μm, (b,c,d,e,f) 5 μm, and (g,h,i, j) 1 mm].

**Figure 3:**
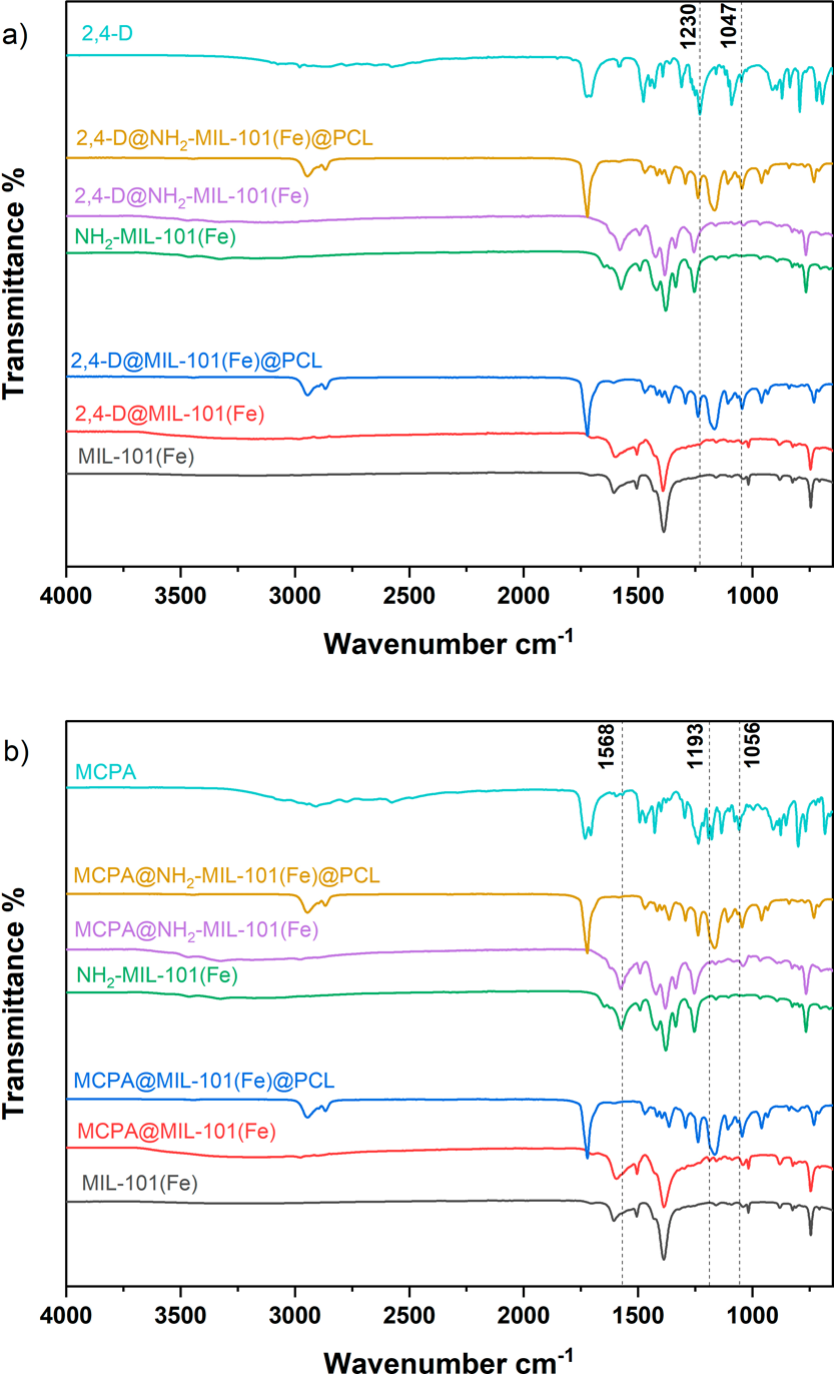
FTIR spectra of (a) MIL-101(Fe), NH_2_-MIL-101(Fe) (pristine and loaded with 2,4-D), their PCL composites, and 2,4-D (b) MIL-101(Fe), NH_2_-MIL-101(Fe) (pristine and loaded with MCPA), their PCL composites, and MCPA.

The FTIR spectra of MIL-101(Fe) and NH_2_-MIL-101(Fe) are consistent with spectra reported in previous literature.^32,36−39^ The peak between 1580 and 1660 cm^–1^ can be attributed to the symmetric C=O stretching of carboxylate groups coordinated to iron. Peaks at 1502 and 1580 cm^–1^ originate from the C=C stretching vibrations of the phenyl rings in the linker, while the peak at 770 cm^–1^ accounts for the C–H bending vibrations of the organic linker.

Compared to MIL-101(Fe), the amino-functionalized MIL-101(Fe) shows two characteristic peaks at 1255 and 1329 cm^–1^. These peaks account for the C–N stretching and N–H bending of the amino group in 2-amino-1,4-benzenedicarboxylic acid.^36^ Broad peaks at 3469 and 3327 cm^–1^ in NH2-MIL-101(Fe) are attributed to the symmetric and asymmetric N–H bond stretching of primary amines, respectively.^36,39^ For the 2,4-D@MOF composite, bands at 1047 and 1230 cm^–1^ indicate the presence of 2,4-D, corresponding to the C–O–C stretching of the ester group (Figure 3a).

In the case of MCPA@MIL-101(Fe), MCPA@NH_2_-MIL-101(Fe), and their PCL composites, bands around 1193 and 1056 cm^–1^ can be observed due to C–O stretching (Figure 3b). The spectral band at 1568 cm^–1^, attributed to the C=C stretching in MCPA, exhibits broadening in the MCPA@MOFs. This indicates a change in the chemical environment of MCPA within the MOF due to interactions with the MOF framework, indicating successful MCPA loading. When the herbicides@MOF is incorporated into the PCL matrix, the peaks originating from the herbicides@MOFs are negligible compared to the peaks originating from the pure PCL (Figure S4). This is because the quantity of herbicide@MOFs is relatively very low compared to the amount of PCL in the composite, and therefore, the peaks corresponding to the herbicide@MOFs are masked by the peaks of the PCL. This finding also correlates with the PXRD data.

The thermal stability of all MOFs was studied under N_2_ gas in detail, and the TGA plots are provided (Figure S5). The decomposition of MOFs shows three stages of weight loss. The first weight loss below 150 °C is due to the removal of water or solvent molecules occupying the pores and surfaces of the frameworks.^32^ The second weight loss, observed at temperatures of 150–320 °C, is due to the decomposition of the free organic structure present in the frameworks.^40^ Subsequently, the loss after 320 °C is attributed to the decomposition of the linker as a result of the breakdown of the framework.^41^

In the case of the herbicide-loaded samples, an additional weight loss stage was observed between 130 and 300 °C, which was attributed to volatilization and degradation of herbicides.^41,42^ The final weight loss occurred beyond 300 °C, indicating the complete degradation resulting from linker degradation, leading to the collapse of the MOF structure.^41^

The TGA analyses of MOF-loaded PCL composites showed that PCL started to decompose at approximately 200 °C, leading to a substantial weight loss of ∼90% for all of the composites. The weight loss between 200 and 400 °C is due to the degradation of the herbicides and the organic structure of MOFs present in the PCL matrix. Beyond 400 °C, MOF@PCL composites show a small weight loss due to the collapse of the MOF framework in the PCL matrix, reaching a residual weight of ∼5% for MOF loaded with 2,4-D and MCPA at 700 °C. This residual weight can be attributed to the presence of inorganic iron oxide resulting from the degradation of the MOFs.

**Figure 4:**
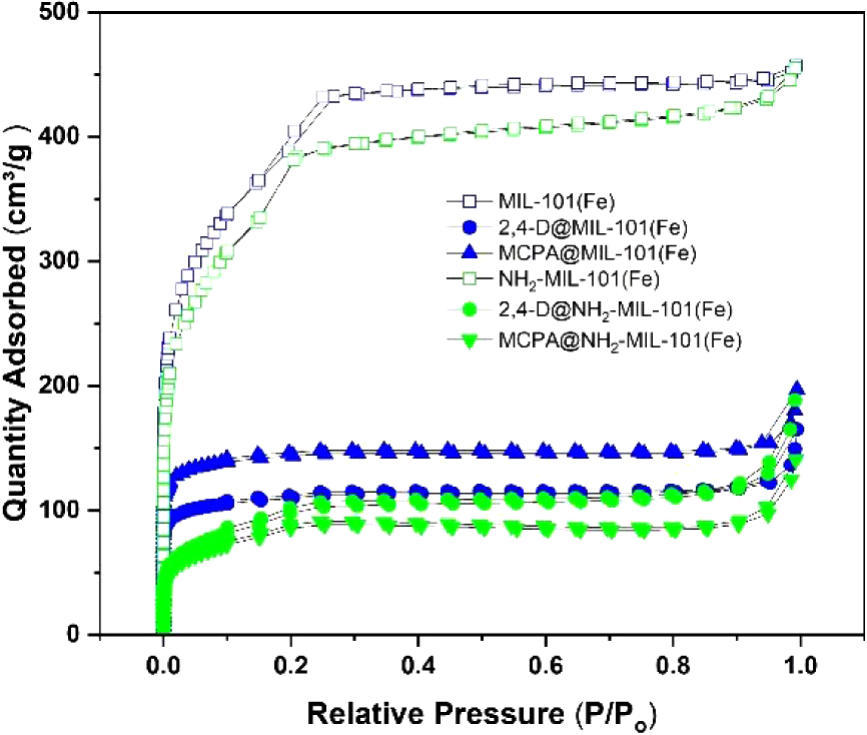
Nitrogen adsorption–desorption isothermal cycles at 77 K of Fe-MOF samples used in this work.

N_2_ gas sorption experiments were carried out at 77 K to determine the surface area, pore volume, and pore size distribution of the samples. The adsorption–desorption N_2_ isotherms of the herbicide-loaded MOFs were compared against those of MIL-101(Fe) and NH_2_-MIL-101(Fe) (Figure 4).

**Figure 5:**
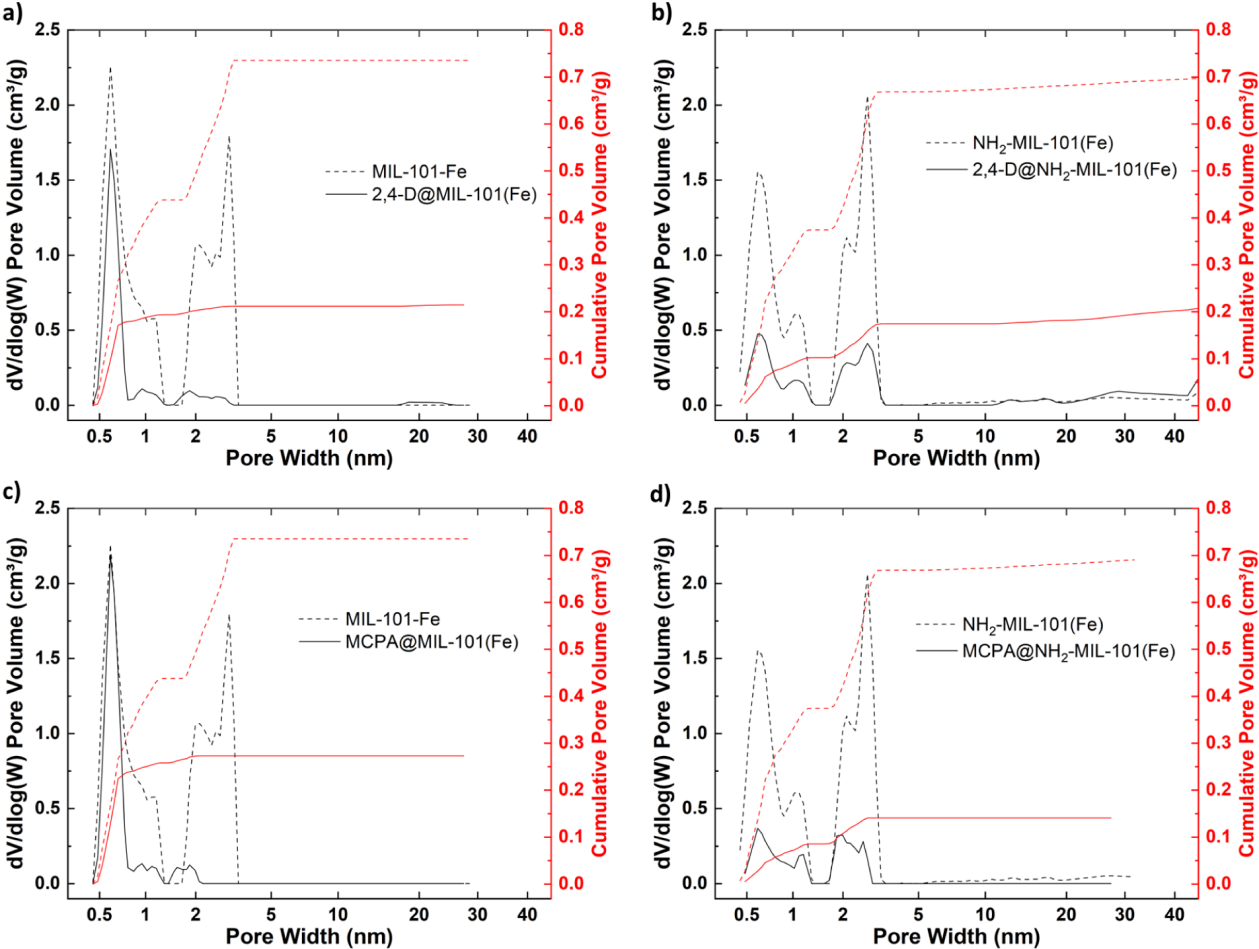
(a–d) Pore size distribution and cumulative pore volume of MOF samples, from 77 K N_2_ sorption data fitted with a Tarazona cylindrical pore NLDFT model.

As classified by IUPAC,^43^ both pristine MOFs and herbicide-loaded NH_2_-MIL-101(Fe) MOFs exhibit type IV(b) isotherms, characteristic of materials exhibiting both micro and mesopores <4 nm in width. Both herbicide-loaded MIL-101(Fe) MOFs exhibit type I isotherms, indicating materials predominantly composed of micropores <2 nm. This is consistent with the pore size distribution calculated for the native pristine samples, as illustrated in Figure 5. In comparison to the respective pristine MOFs, the herbicide-loaded MOFs exhibited a reduced quantity of adsorbed N_2_, indicating decreased porosity. This reduction is further evidenced by the diminished surface areas (SAs) and total pore volumes (TPVs) observed for the MOF samples, as summarized in Table 1. Surface area measurements were performed for each sample and fitted using the Rouquerol method^44^ with a positive y-intercept and the highest R^2^ values (Figure S6).

**Table 1:** Pore Characteristics of the Iron-Based MOF Samples

Sample ID	BET surface area (m^2^g^–1^)	Total pore volume (TPV)^a^ (cm^3^g^–1^)	Micro and mesopore volume^b^ (cm^3^g^–1^)	Pore volume reduction (%)^c^
MIL-101(Fe)	1397 ± 2.03^#^	0.69	0.74	--
NH_2_-MIL-101(Fe)	1300 ± 3.9	0.67	0.67	--
2,4-D@MIL-101(Fe)	424 ± 0.5	0.21	0.21	69
MCPA@MIL-101(Fe)	562 ± 0.4	0.26	0.27	62
2,4-D@NH_2_-MIL-101(Fe)	345 ± 0.9	0.20	0.17	70
MCPA@NH_2_-MIL-101(Fe)	308 ± 1.8	0.15	0.14	77

a Single-point adsorption total pore volume of pores taken at *P*/*P*_o_ = 0.94.

b DFT cumulative pore volume of pores <52 Å.

c With respect to the pristine MOFs; # the standard deviation for the BET surface area is from 3Flex fitting calculation.

Comparing the SA and TPV of 2,4-D@MOFs and MCPA@MOFs to MIL-101(Fe) and NH_2_-MIL-101(Fe), it was found that (a) 2,4-D@MIL-101(Fe) observed a 70% reduction in SA and 69% reduction in TPV in comparison to MIL-101(Fe); (b) MCPA@MIL-101(Fe) observed a 60% decrease in SA and 62% reduction in TPV in comparison to MIL-101(Fe); (c) 2,4-D@NH_2_-MIL-101(Fe) observed a 73% reduction in SA and 70% increase in TPV in comparison to NH_2_-MIL-101(Fe); and (d) MCPA@NH_2_-MIL-101(Fe) observed a 76% decrease in SA and a 77% decrease in TPV in comparison to NH_2_-MIL-101(Fe). The reduction in the total pore volume and surface area could be due to 2,4-D and MCPA occupying the available pore volume or losses in the MOF crystallinity during the loading process, as was observed in the PXRD data (Figure 1).

To further elucidate the effect of herbicide infiltration, pore size distributions and cumulative pore volumes were calculated by fitting the isotherms to DFT models. These results are compared against pristine MOFs (Figure 5). Changes in the pore network and available volume within the individual pores of the samples reveal whether they are occupied/blocked with molecules or if any changes in pore sizes occurred during processing.

Across all samples, the most significant reduction in available pore volume was observed in the mesopores, with up to a 97% reduction in the available pore volume in the 30.6 Å pore for MIL-101(Fe) after the introduction of 2,4-D. A significant reduction in micropore volume was observed for the amino-functionalized MOF (Table S2). However, in MIL-101(Fe), the smallest pore (5.9 Å) showed no significant losses, indicating that the pesticide molecules did not occupy these pores.

In addition, the introduction of herbicides into the MOFs led to changes in the width of available pores (Table S3), indicating alterations to the MOF structure. The most significant changes were observed for MCPA@MIL-101(Fe), with up to a 40% reduction in the mesopore width. The results suggest that herbicides chiefly accumulate in the mesopores of the samples, reducing the available pore volume for nitrogen molecules to occupy. The presence of amino-functional groups in the MOFs appears to enhance the infiltration of pesticide molecules into the micropores, thus increasing the storage capacity of the MOF.

### 3.2 Loading and Release Study

PXRD, FTIR, SEM, and elemental mapping confirmed the successful loading of 2,4-D and MCPA into the two MOFs. The loading capacity for herbicides in the MOFs was estimated by suspending the loaded MOFs with distilled water and sonicating for 60 min. The loading capacities were found to be 18.06% for 2,4-D@MIL-101(Fe) and 21.51% for MCPA@MIL-101(Fe). In contrast, the loading capacities were 26.61% for 2,4-D@NH_2_-MIL-101(Fe) and 23.32% for MCPA@NH_2_-MIL-101(Fe). The higher loading capacity for the amino-functionalized MOF can be attributed to hydrogen bonding between the NH_2_-group of NH_2_-MIL-101(Fe) and the carboxylate and chloride groups of 2,4-D and MCPA.

**Figure 6:**
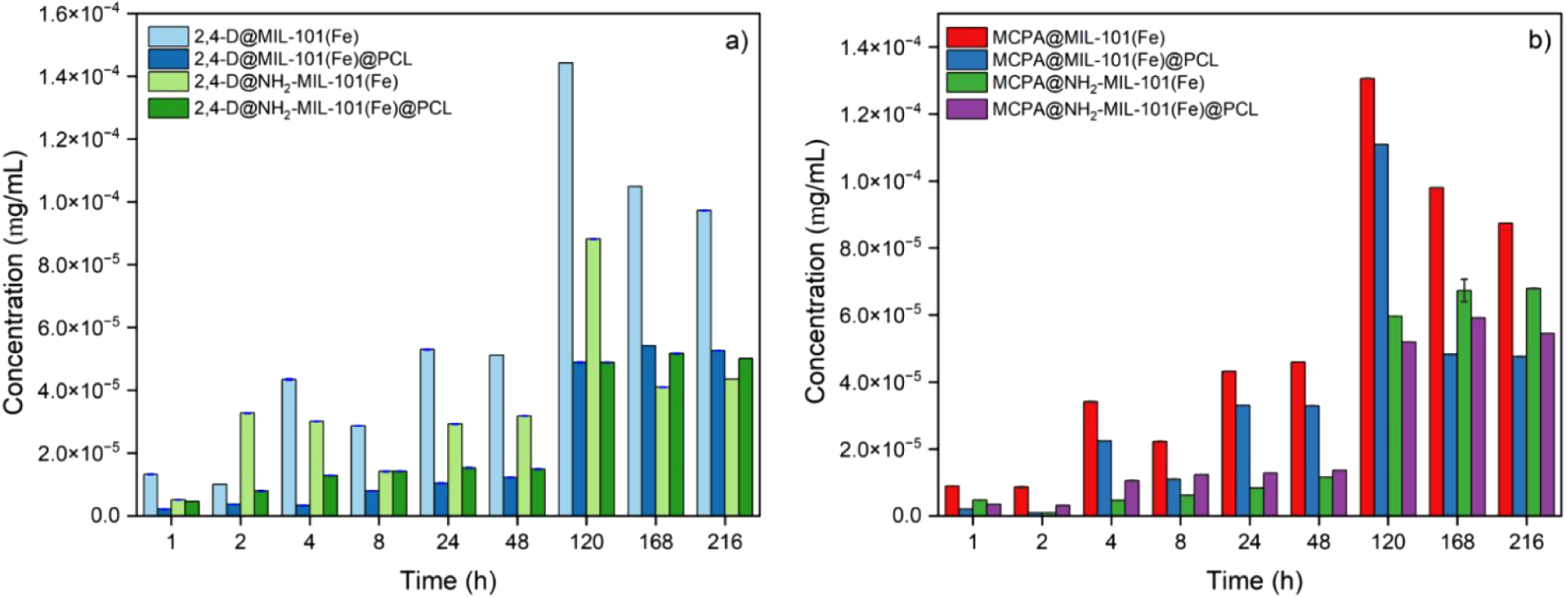
Release study of (a) 2,4-D and b) MCPA from MIL-101(Fe), NH_2_-MIL-101(Fe), and their PCL composites in water over time.

This is further supported by the greater loss in pore volume for the loaded NH_2_-MIL-101(Fe). All sets of 2,4-D and MCPA-loaded MOFs and their PCL composites were immersed in 30 mL of distilled water at room temperature for 216 h. The filtered aliquots were analyzed using a UV–visible spectrophotometer to quantify the amounts of 2,4-D and MCPA released from the MOFs and their PCL composite. As shown in Figure 6, for both the 2,4-D and MCPA-loaded MOFs, the release of the herbicide in water was faster compared to the PCL composites, with maximum values of 1.44 × 10^–4^ mg mL^–1^ and 8.82 × 10^–5^ mg mL^–1^ for 2,4-D@MIL-101(Fe) and 2,4-D@NH_2_-MIL-101(Fe), respectively. For MCPA@MIL-101(Fe) and MCPA@NH2-MIL-101(Fe), the values were 1.31 × 10^–4^ and 6.79 × 10^–5^ mg mL^–1^, respectively. The higher release of herbicides from both of the MOFs may be because of the herbicides binding loosely to the MOF, allowing for faster diffusion from the pores. Furthermore, comparing NH_2_-MIL-101(Fe) to MIL-101(Fe) loaded with 2,4-D and MCPA, the herbicide-loaded MIL-101(Fe) shows higher release rates beyond 120 h. This difference can be attributed to MIL-101(Fe) providing fewer binding sites for herbicides compared with NH_2_-MIL-101(Fe), facilitating faster diffusion from its structure.

However, for NH_2_-MIL-101(Fe), the herbicide release was slightly reduced due to weak H-bonding between the NH_2_- group of NH_2_-MIL-101(Fe) and the carboxylate group of 2,4-D and MCPA. Recent release studies of 2,4-D and MCPA using Zr-based MOFs (UiO-66 and UiO-66-NH_2_) showed that, in general, amino-functionalized UiO-66 exhibits a slower release than UiO-66 due to hydrogen bond interactions between the carboxylate group of the 2,4-D and −NH_2_ groups of UiO-66-NH_2_.^26,39^

When the herbicide@MOFs were incorporated into the PCL matrix, the herbicide release was more controlled. For instance, the amount of 2,4-D released from 50 mg of MOF-loaded PCL composite (containing 5 mg of herbicide@MOFs) was 5.42 × 10^–5^ mg mL^–1^ for 2,4-D@MIL-101(Fe)@PCL and 5.17 × 10^–5^ mg mL^–1^ much more controlled compared to the powder form (1.44 × 10^–4^ mg mL^–1^ and 8.82 × 10^–5^ mg mL^–1^ for 2,4-D@MIL-101(Fe) and 2,4-D@NH_2_- MIL-101(Fe), respectively. Similar patterns were observed for MCPA in the iron-MOF PCL composites, with release values of 4.83 × 10^–5^ mg mL^–1^ and 5.91 × 10^–5^ mg mL^–1^ for MCPA@MIL-101(Fe)@PCL and MCPA@NH_2_-MIL-101(Fe)@PCL, respectively. In both cases, MIL-101(Fe) showed faster herbicide release compared to NH_2_-MIL-101(Fe). This correlates to the hypothesis that the carboxylate group in 2,4-D and MCPA interacts with the amino group of the NH_2_-MIL-101(Fe) via hydrogen bonding, leading to a slower release of the herbicides.

A decrease in release was observed after 120 h for the herbicide-loaded MOFs. As discussed above, the structure of the MOFs degrade over time with the possible formation of iron oxo and hydroxo complexes, which is evident from the PXRD patterns of the loaded MOFs, where numerous small peaks characteristic of α-FeO(OH) were observed.^45^ From earlier studies, α-FeO(OH) is also reported to be one of the best sorbents for phenoxy acid herbicides (in this case, 2,4-D and MCPA).^46^ Thus, the decrease of the release after 120 h is potentially due to the interaction of α-FeO(OH) with the herbicides to form outer-sphere and inner-sphere complexes, as reported by Kersten et al.^46^

**Figure 7:**
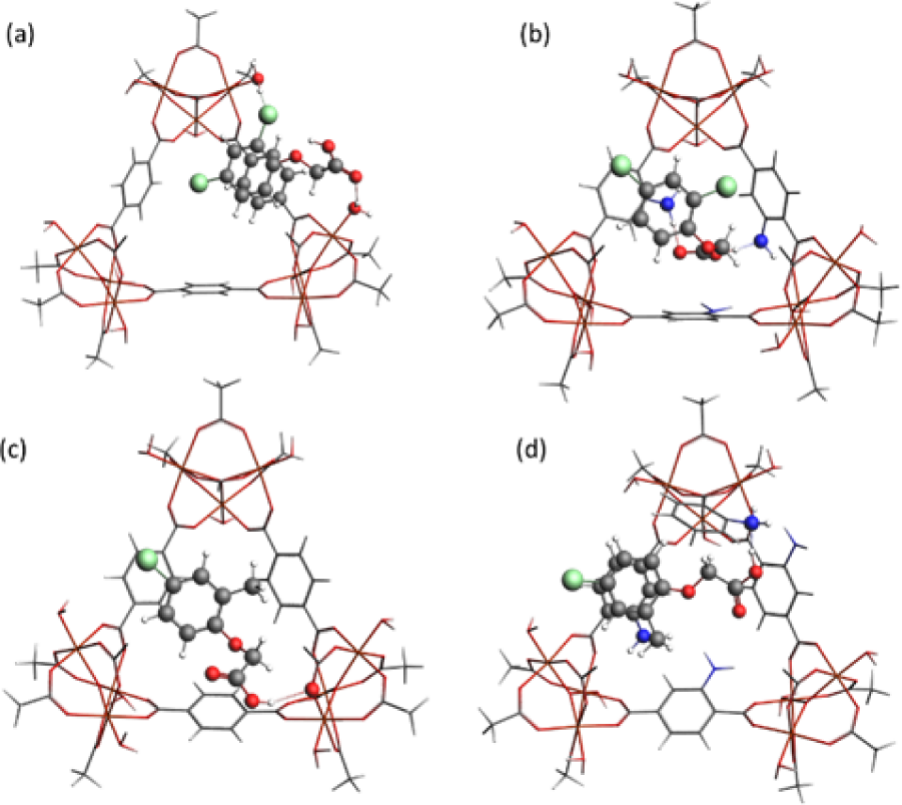
Model showing the interaction of 2,4-D with (a) MIL-101(Fe), (b) NH_2_-MIL-101(Fe), and MCPA with (c) MIL-101(Fe) and (d) NH_2_-MIL-101(Fe).

To understand the interactions between the MOFs and herbicides in more detail, computational studies were performed (see Supporting Information for details). The results indicate the formation of hydrogen bonds between the amino group of NH_2_-MIL-101(Fe) and the carboxylate group of the 2,4-D and MCPA molecules, consistent with the slower release observed for the NH_2_-MIL-101(Fe). The binding energy was found to be −203.99 kJ mol^–1^ for the interaction between 2,4-D and NH_2_-MIL-101(Fe), which is 45% higher than the binding energy for the interaction between 2,4-D and MIL-101(Fe) (Figure 7). Similarly, the binding energy was −198.73 kJ mol^–1^ for the interaction between MCPA with NH_2_-MIL-101(Fe), 22% higher than for MIL-101(Fe). The higher binding energy for 2,4-D with NH_2_-MIL-101(Fe) compared to the interaction of MCPA with the same Fe-MOF is likely to be due to more extensive interactions of the 2,4-D molecule via two −Cl groups and a −COOH group, as opposed to MCPA, which has only one chloride group. The two chloride groups, along with the −COOH group, actively form hydrogen bonds with the amino group of the linker.

For 2,4-D@MIL-101(Fe), a π-stacking distance of 3.42 Å was observed between the linker of MIL-101(Fe) and the phenyl ring of 2,4-D. The H-bond lengths were calculated as 2.062 Å for Cl···HOH (metal cluster cap) and 1.866 Å for H···HOH (metal cluster cap). In contrast, no π-stacking was observed for MCPA@MIL-101(Fe), with a hydrogen bond length of 2.010 Å between MCPA and MIL-101(Fe). In the case of NH_2_-MIL-101(Fe), a slight offset π-stacking of 3.87 Å was observed between the linker of NH_2_-MIL-101(Fe) and the phenyl ring of 2,4-D. The H-bond lengths were calculated as 2.171 Å for O···HN-H (linker of the MOF) and 1.425 Å for OH···NH_2_ (linker of the MOF). For MCPA@NH_2_-MIL-101(Fe), a π-stacking distance of 3.44 Å was observed between the linker and the phenyl ring, with a H-bond length of 1.475 Å between MCPA’s OH···NH_2_(linker).

These results indicate that NH_2_-MIL-101(Fe) uses more H-bonding and π-stacking interactions to tightly bind the herbicides to the pore walls. However, in the case of MCPA@MIL-101(Fe), MCPA is loosely held, occupying the center of the pore and blocking it.

## 4 Conclusions

This study has shown potential applications of two iron-based MOFs, namely, MIL-101(Fe) and NH_2_-MIL-101(Fe), for the sustainable delivery of two widely used herbicides, 2,4-D and MCPA. Comprehensive characterizations confirmed the synthesis of MIL-101(Fe) and NH_2_-MIL-101(Fe) and loading of both MOFs with the two herbicides. Despite high herbicide loading capacities, PXRD revealed broadening of diffraction peaks, which was attributed to the partial degradation and loss of crystallinity of the MOFs during the loading process. It should be noted that, in contrast to many other applications of MOFs, degradation of the MOFs is a desired outcome toward the end of their use in agrochemical applications. The herbicide-loaded MOFs were incorporated into biodegradable polycaprolactone (PCL) membranes to develop hybrid composites. The release rates of 2,4-D and MCPA from both MIL-101(Fe) and NH_2_-MIL-101(Fe), as well as their respective PCL composites, were thoroughly investigated. The results indicate a controlled and gradual release of the herbicides when incorporated into PCL, suggesting sustainable and long-lasting release profiles suitable for agricultural applications. These MOF-polymer composites hold promise for the controlled and sustainable delivery of pesticides and other agrochemicals, thereby preventing their overuse. Moreover, the use of the iron-based MOFs not only contributes to the controlled release of herbicides but also can be a potential source of iron as a micronutrient for plants upon degradation. This dual functionality can potentially enhance the environmental friendliness and sustainability of the composites. Further studies are currently underway to understand the time-dependent degradation of these MOFs and their effect on broadleaf weeds and plant growth.

## Supporting Information Available

Additional optical and SEM images, elemental mapping, PXRD plots, IR spectra, BET and pore size distribution plots, TGA plots, and DFT data (PDF)

## Abbreviations

2,4-D: 2,4-dichlorophenoxyacetic acid
BET: Brunauer–Emmett–Teller
IR: infrared
MCPA: 2-methyl-4-chlorophenoxyacetic acid
MOF: metal–organic framework
PCL: polycaprolactone
PXRD: powder X-ray diffraction
SEM: scanning electron microscope
TGA: thermogravimetric analysis
