# Supplementary material for: Iron-Based Metal–Organic Frameworks and Their Polymer Composites for Sustainable Delivery of Herbicides
Source: ACS Omega. 2025 Feb 26;10(9):9051–61. doi: 10.1021/acsomega.4c07972 (PMC11904714; doi:10.1021/acsomega.4c07972)
Supplement: Supplementary file 1 — ao4c07972_si_001.pdf [file ao4c07972_si_001.pdf]

Supporting Information

Iron based Metal-organic Frameworks and their Polymer Composites for Sustainable Delivery of Herbicides

Parimal C. Bhomick,[a,b,c,d] Evdokiya H. Ivanovska,[e] Lila A. M. Mahmoud,[f] Huan V. Doan,[d] Lui R. Terry,[a,g] Matthew A. Addicoat,[h] Jemma L. Rowlandson,[c] Sebastien Rochat,[i] Valeska P. Ting,[a,d]* Sanjit Nayak[a,g]*

a. Bristol Composites Institute, Queen's Building, University of Bristol, University Walk, Bristol BS8 1TR, U.K.
b. Department of Chemistry; Nagaland University, Lumami, Nagaland, IN 798627
c. School of Electrical, Electronic and Mechanical Engineering, University of Bristol, Queen's Building, University Walk, Bristol BS8 1TR, U.K.
d. Research School of Chemistry, Australian National University, Canberra, ACT, AU 2601
e. School of Archaeological and Forensic Sciences, University of Bradford, Bradford, BD7 1DP, U.K.
f. School of Chemistry, University of Bristol, BS8 1TS, Bristol BS8 1TS, U.K.
g. School of Civil, Aerospace and Design Engineering, Queen's Building, University of Bristol, University Walk, Bristol BS8 1TR, U.K.
h. School of Science and Technology, Nottingham Trent University, Clifton Lane, Nottingham NG11 8NS, U.K.
i. School of Engineering Mathematics and Technology, University of Bristol, Bristol BS8 1TR, U.K.

E-mail: s.nayak@bristol.ac.uk (SN), valeska.ting@anu.edu.au (VPT)

Table of Content

Figure S1. MIL-101(Fe) and NH_2-MIL-101(Fe) loaded with 2.4-D and MCPA based PCL composites

Figure S2. PXRD of 2,4-D, MCPA and PCL

Figure S3. Elemental Mapping of (a-c) 2,4-D@MIL-101(Fe)@PCL (d-f) 2,4-D@ NH_2-MIL-101(Fe)@PCL (g-i) MCPA@MIL-101(Fe)@PCL (j-l) MCPA@NH_2-MIL-101(Fe)@PCL

Figure S4. FTIR Spectrum of polycaprolactone (PCL)

Figure S5. TGA plots of (a) pristine, 2,4-D and MCPA MIL-101(Fe) (b) pristine, 2,4-D and MCPA loaded NH_2-MIL-101(Fe) (c) PCL, 2,4-D and MCPA loaded MIL-101(Fe) PCL composites (d) PCL, 2,4-D and MCPA loaded NH_2-MIL-101(Fe) PCL composites

Figure S6. BET Plot of each Fe-MOF sample, displaying correlation function, slope, Y-intercept and C values.

Table S1 Standard deviation of fit of Pore size distribution models used (DFT – N_2 Cylindrical – Tarazona – Esf = 30K)

Table S2 Percentage change in available pore volume compared to pristine MOF pores of MIL-101(Fe) / NH_2-MIL-101(Fe) (accounting for pore size changes)

Table S3 Percentage change in pore width compared to pristine MOF pores of MIL-101(Fe) / NH_2-MIL-101(Fe)

Loading of 2,4-D and MCPA

Computational Method

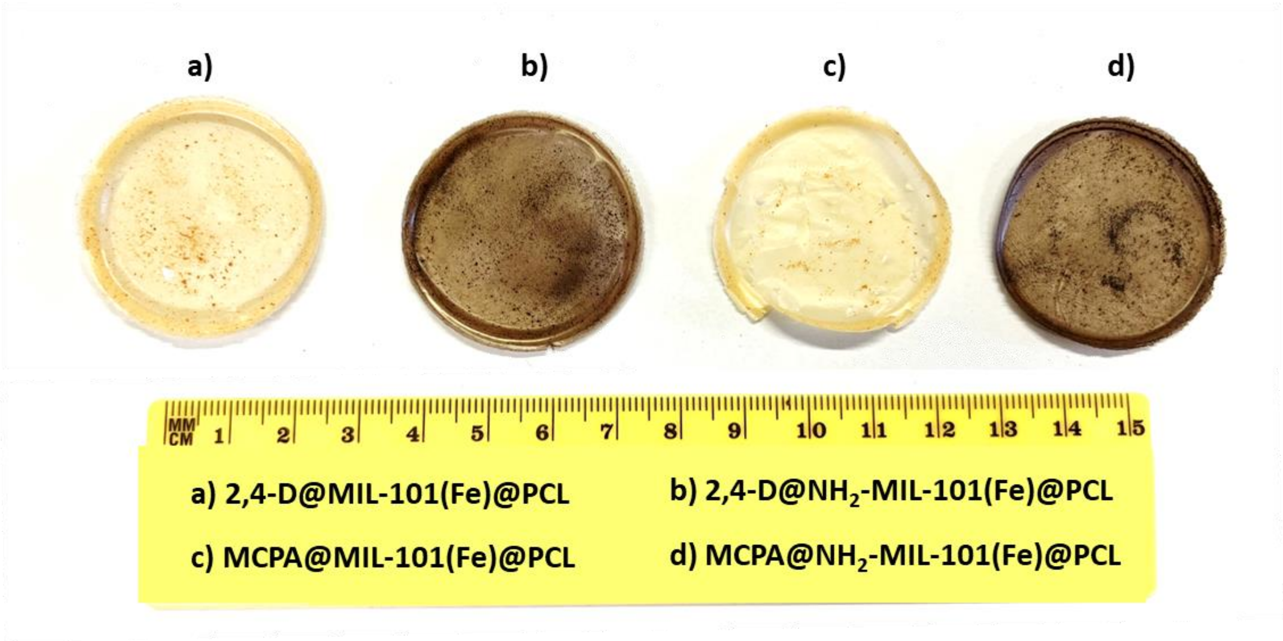

Figure S2. MIL-101(Fe) and NH_2-MIL-101(Fe) loaded with 2.4-D and MCPA based PCL composites: a) 2,4-D@MIL-101(Fe)@PCL, b) 2,4-D@NH_2-MIL-101(Fe)@PCL, c) MCPA@MIL-101(Fe)@PCL, and d) MCPA@NH_2-MIL-101(Fe)@PCL

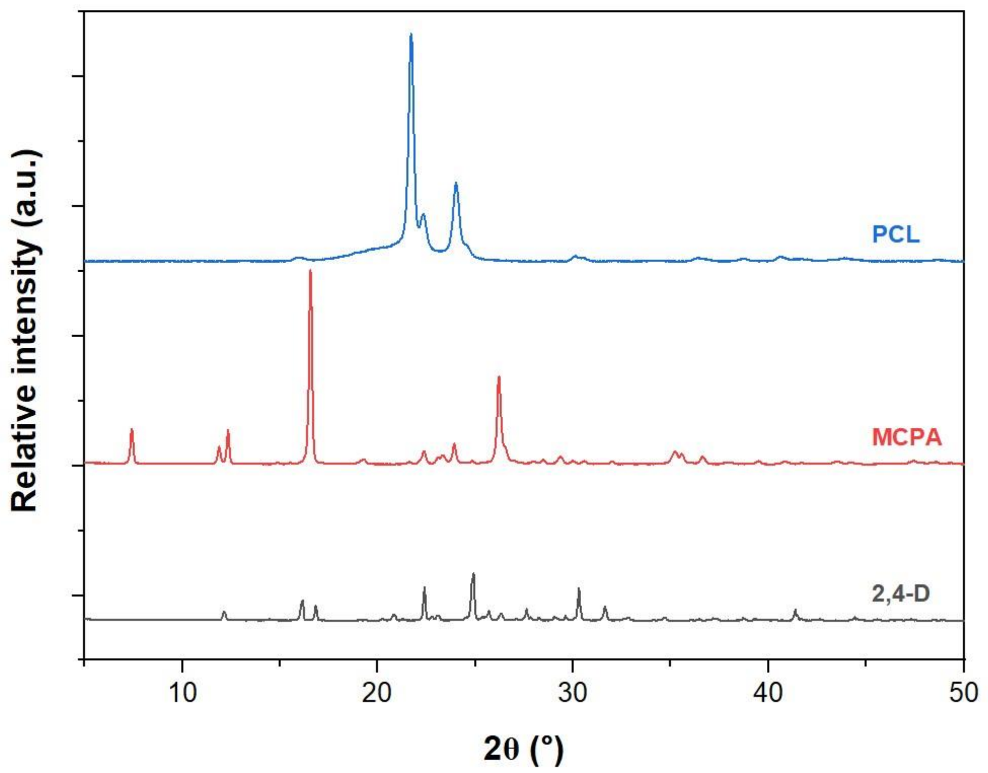

Figure S2. PXRD of 2,4-D, MCPA and PCL

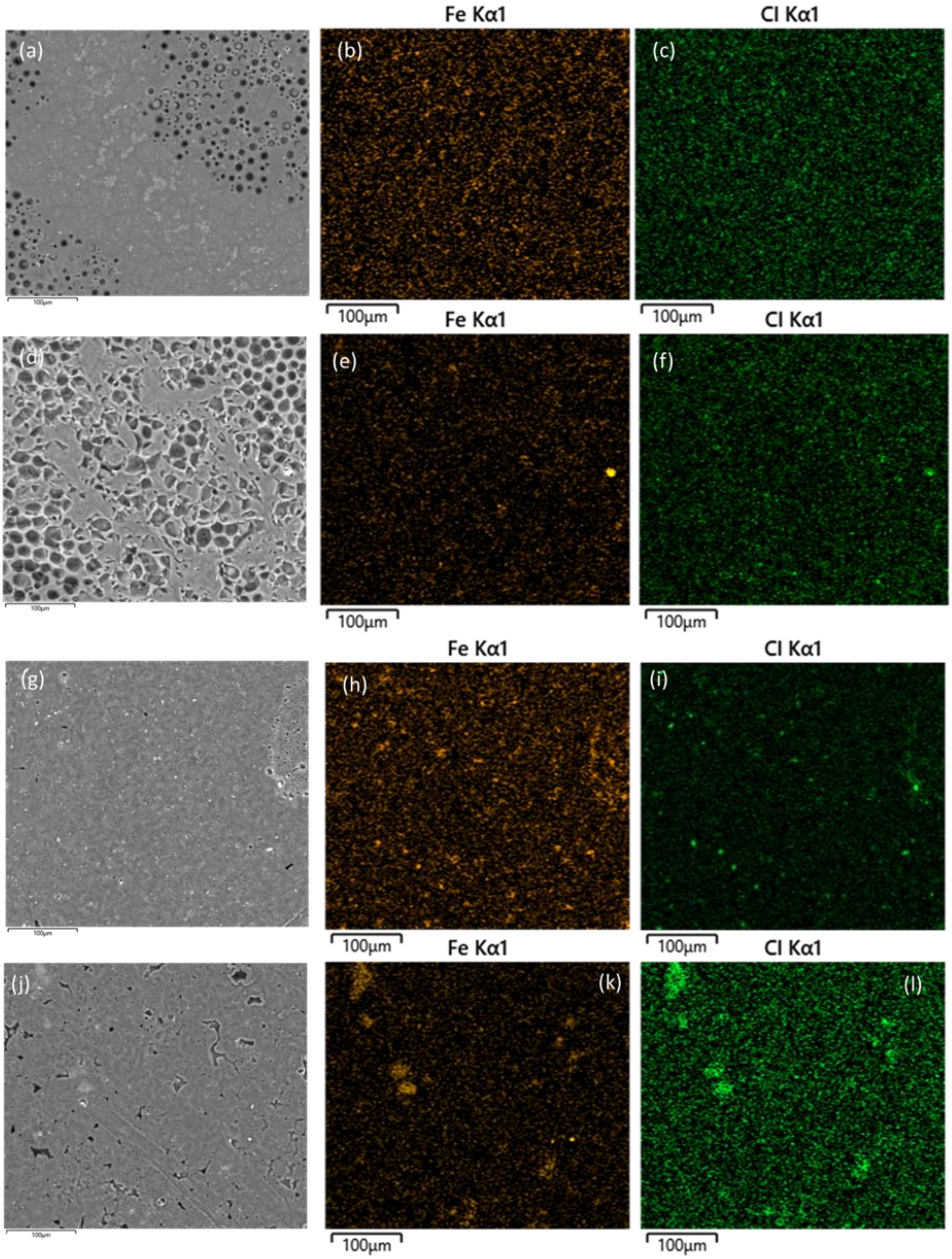

Figure S3. Elemental Mapping of (a-c) 2,4-D@MIL-101(Fe)@PCL (d-f) 2,4-D@ NH_2-MIL-101(Fe)@PCL (g-i) MCPA@MIL-101(Fe)@PCL (j-l) MCPA@NH_2-MIL-101(Fe)@PCL

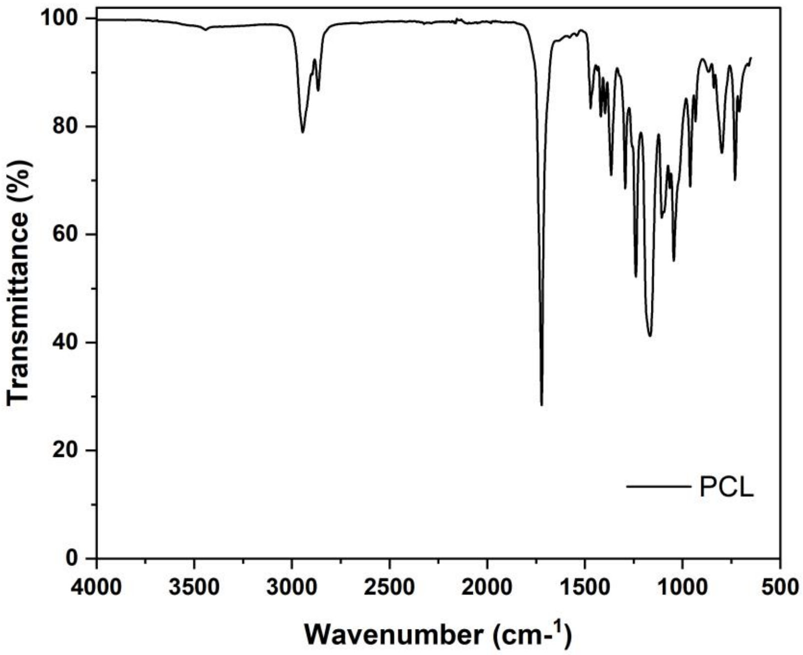

Figure S4. FTIR Spectrum of polycaprolactone (PCL)

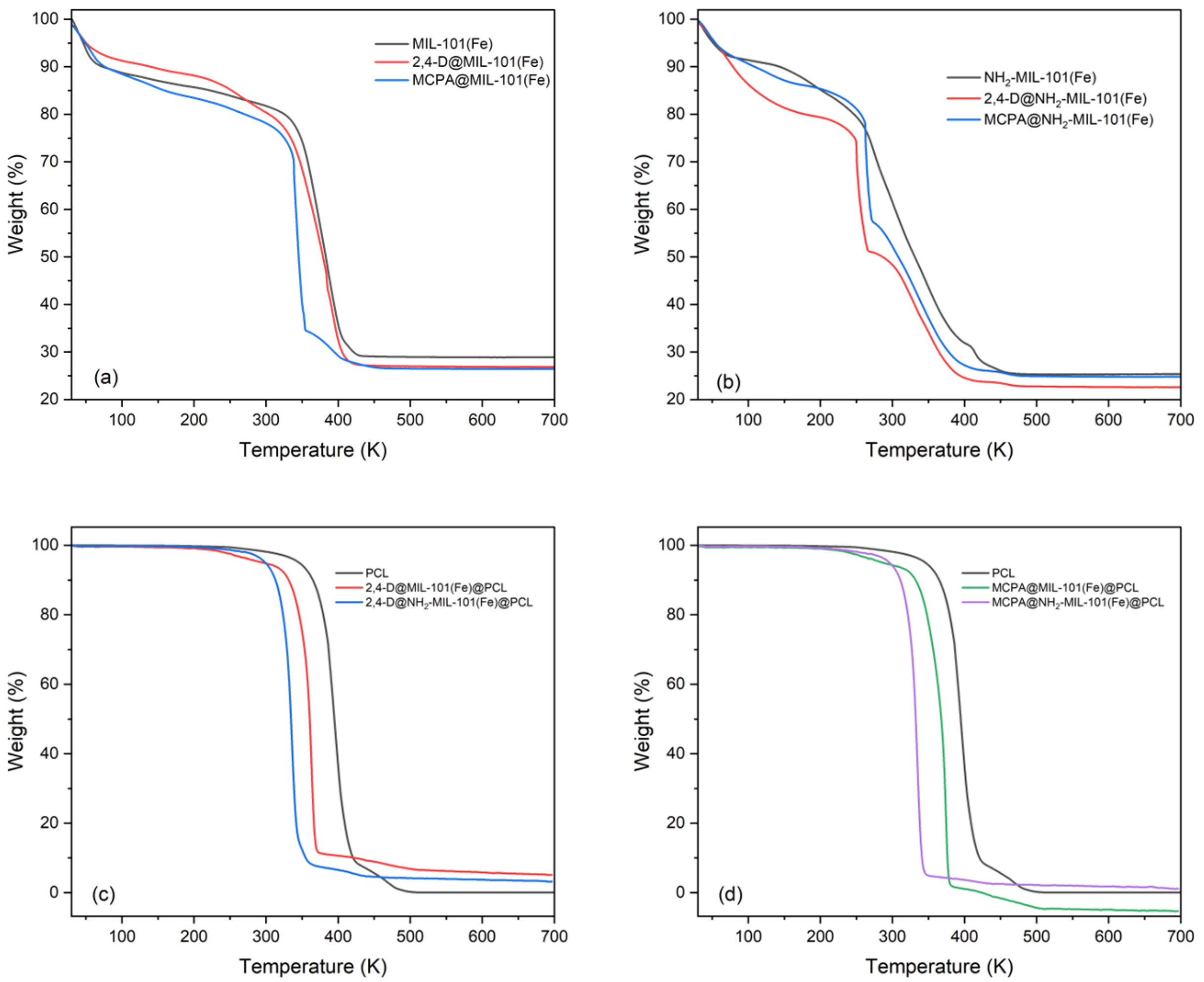

Figure S5. TGA plots of (a) pristine, 2,4-D and MCPA MIL-101(Fe) (b) pristine, 2,4-D and MCPA loaded NH_2-MIL-101(Fe) (c) PCL, 2,4-D and MCPA loaded MIL-101(Fe) PCL composites (d) PCL, 2,4-D and MCPA loaded NH_2-MIL-101(Fe) PCL composites

BET Plots for Fe-MOF samples.

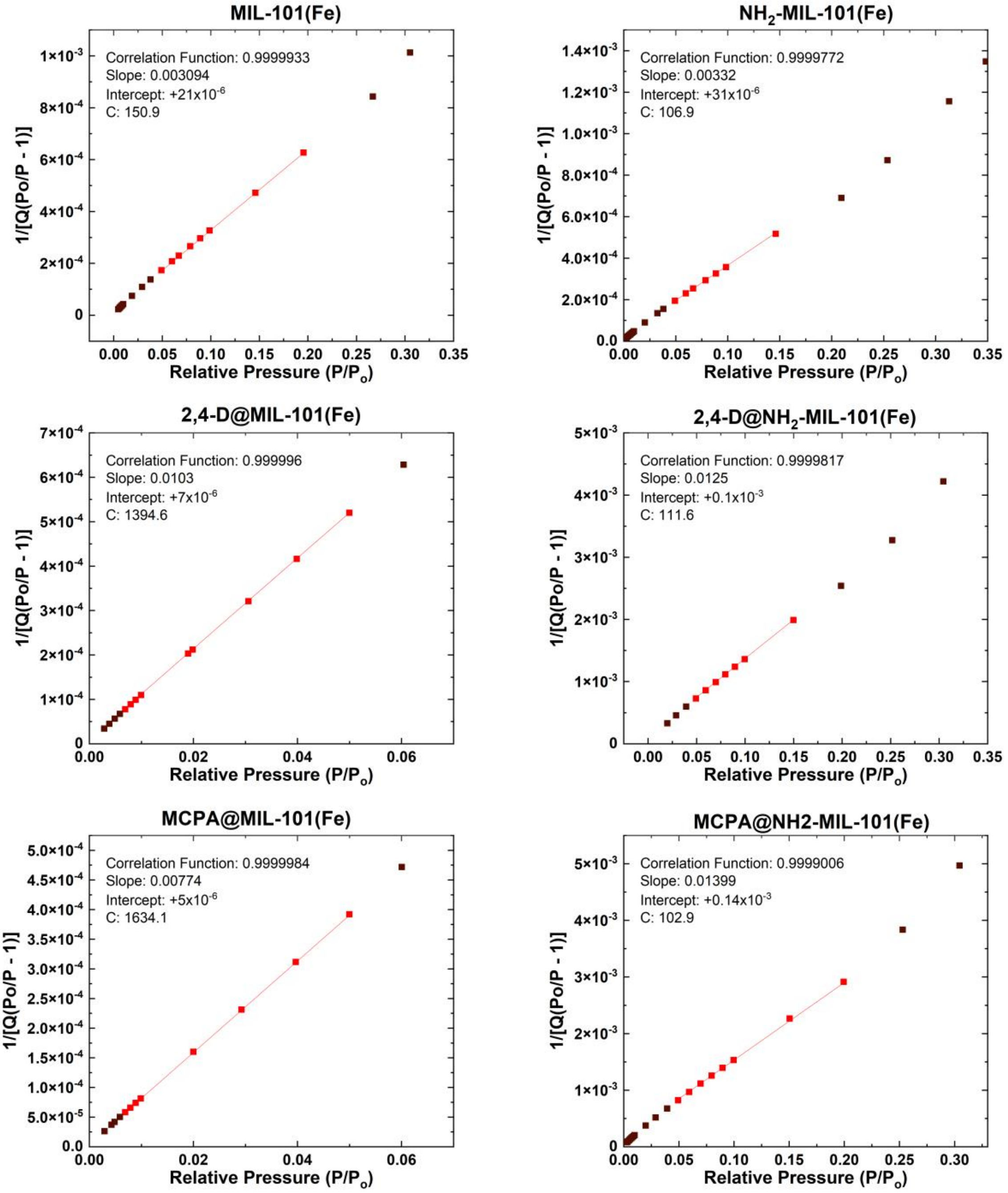

Figure S6. BET Plot of each Fe-MOF sample, displaying correlation function, slope, Y-intercept and C values.

Table S1: Standard deviation of fit of Pore size distribution models used (DFT – N_2 Cylindrical – Tarazona – Esf = 30K)

Sample	PSD - standard deviation of fit (cm^3/g)
MIL-101(Fe)	3.35760
MCPA@MIL-101(Fe)	1.27924
2,4-D@MIL-101(Fe)	0.80300
NH_2-MIL-101(Fe)	2.61515
MCPA@NH_2-MIL-101(Fe)	1.80099
2,4-D@NH_2-MIL-101(Fe)	0.68102

Table S2: Percentage change in available pore volume compared to pristine MOF pores of MIL-101(Fe) / NH_2-MIL-101(Fe) (accounting for pore size changes)				
samples	Percentage change in available pore volume compared to pristine MOF pores of MIL-101-Fe / MIL-101-Fe-NH_2			
	Micropores (**Å**)		Mesopores (**Å**)	
	5.9 / 5.9	9.5 / 10.9	20.9 / 20.9	30.6 / 27.4
MMCPA@MIL-101(Fe)	-3%	-80%	-90%	-93%
M24D@MIL-101(Fe)	-25%	-83%	-91%	-97%
MMCPA@NH_2-MIL-101(Fe)	-76%	-68%	-71%	-86%
M24D@NH_2-MIL-101(Fe)	-69%	-72%	-75%	-80%

Table S3: Percentage change in pore width compared to pristine MOF pores of MIL-101(Fe) / NH_2-MIL-101(Fe)				
samples	Percentage change in pore width compared to pristine MOF pores of MIL-101-Fe / MIL-101-Fe-NH_2			
	Micropores (**Å**)		Mesopores (**Å**)	
	5.9 / 5.9	9.5 / 10.9	20.9 / 20.9	30.6 / 27.4
MMCPA@MIL-101(Fe)	0	0	-25%	-40%
M24D@MIL-101(Fe)	0	0	-12%	-10%
MMCPA@NH_2-MIL-101(Fe)	0	+7%	-5%	-5%
M24D@NH_2-MIL-101(Fe)	0	-6%	0	0

Loading of 2,4-D and MCPA:

A stock solution was prepared by dissolving 1.54 g of 2,4-D and 1.4 g of MCPA in 100 mL of ethanol, respectively. For the loading process, 0.05 g of each pristine MOF sample was weighed into separate glass vials, and 10 mL of the stock solution was added to each. The suspensions were stirred at 800 rpm for 24 hours. The MOF samples were then recovered by centrifugation, washed three times with ethanol (3 × 10 mL, 20 minutes each at 4600 rpm), and dried in a vacuum oven at 60 °C for 24 hours.

Computational Method:

Coordinates of the tetrahedral pore of MIL-101 were extracted from the crystal structure and methyl capped. Each linker had an amino group added to produce a model of the tetrahedral pore of NH_2-MIL-101. 2,4-D and MCPA molecules were inserted stochastically into both the bare and amino pores using the Kick3 stochastic structure generator.[1] Initial geometries were pre-optimized using UFF4MOF fixing the Fe atoms in their crystallographic positions, before optimization with GFN1-xTB[2] as implemented in AMS2022.[3] Binding energies of 2,4-D and MCPA within the MOF pores were calculated from the final GFN1-xTB optimized geometries by: BE = E(mol@MOF) – [E(MOF) + E(mol)].

[1] M. A. Addicoat, S. Fukuoka, A. J. Page, S. Irle, *J. Comput. Chem.* **2013**, 34, 2591.
[2] S. Grimme, C. Bannwarth, P. Shushkov, *J. Chem. Theory Comput.* **2017**, 13, 1989
[3] AMS **2023**.1, SCM, Theoretical Chemistry, Vrije Universiteit, Amsterdam, The Netherlands, http://www.scm.com
